# Caspase-Dependent HMGB1 Release from Macrophages Participates in Peripheral Neuropathy Caused by Bortezomib, a Proteasome-Inhibiting Chemotherapeutic Agent, in Mice

**DOI:** 10.3390/cells10102550

**Published:** 2021-09-27

**Authors:** Maho Tsubota, Takaya Miyazaki, Yuya Ikeda, Yusuke Hayashi, Yui Aokiba, Shiori Tomita, Fumiko Sekiguchi, Dengli Wang, Masahiro Nishibori, Atsufumi Kawabata

**Affiliations:** 1 Laboratory of Pharmacology and Pathophysiology, Faculty of Pharmacy, Kindai University, 3-4-1 Kowakae, Higashiosaka 577-8502, Japan; maho@phar.kindai.ac.jp (M.T.); takayamiyazaki5@gmail.com (T.M.); 5878.tp.1268@gmail.com (Y.I.); h.429884@gmail.com (Y.H.); 1711610148j@kindai.ac.jp (Y.A.); chiso.bum.230@gmail.com (S.T.); fumiko@phar.kindai.ac.jp (F.S.); 2 Department of Pharmacology, Graduate School of Medicine, Dentistry and Pharmaceutical Sciences, Okayama University, Okayama 700-8558, Japan; dengliwang@okayama-u.ac.jp (D.W.); mbori@md.okayama-u.ac.jp (M.N.)

**Keywords:** bortezomib, high mobility group box 1 (HMGB1), chemotherapy-induced peripheral neuropathy, macrophage, caspase, apoptosis

## Abstract

Given the role of macrophage-derived high mobility group box 1 (HMGB1) in chemotherapy-induced peripheral neuropathy (CIPN) caused by paclitaxel, we analyzed the role of HMGB1 and macrophages in the CIPN caused by bortezomib, a proteasome-inhibiting chemotherapeutic agent used for the treatment of multiple myeloma. Repeated administration of bortezomib caused CIPN accompanied by early-stage macrophage accumulation in the dorsal root ganglion. This CIPN was prevented by an anti-HMGB1-neutralizing antibody, thrombomodulin alfa capable of accelerating thrombin-dependent degradation of HMGB1, antagonists of the receptor for advanced glycation end-products (RAGE) and C-X-C motif chemokine receptor 4 (CXCR4), known as HMGB1-targeted membrane receptors, or macrophage depletion with liposomal clodronate, as reported in a CIPN model caused by paclitaxel. In macrophage-like RAW264.7 cells, bortezomib as well as MG132, a well-known proteasome inhibitor, caused HMGB1 release, an effect inhibited by caspase inhibitors but not inhibitors of NF-κB and p38 MAP kinase, known to mediate paclitaxel-induced HMGB1 release from macrophages. Bortezomib increased cleaved products of caspase-8 and caused nuclear fragmentation or condensation in macrophages. Repeated treatment with the caspase inhibitor prevented CIPN caused by bortezomib in mice. Our findings suggest that bortezomib causes caspase-dependent release of HMGB1 from macrophages, leading to the development of CIPN via activation of RAGE and CXCR4.

## 1. Introduction

Chemotherapy-induced peripheral neuropathy (CIPN) frequently occurs in cancer patients undergoing chemotherapy with platinum agents, taxanes, vinca alkaloids, proteasome inhibitors, etc. CIPN impairs the patients’ quality of life and the dose reduction or discontinuation of the chemotherapy [1,2]. The pathogenesis of CIPN largely remains unclear, and there are presently no effective pharmacological interventions approved for the prevention or treatment of CIPN, although the recent systemic literature reviews have ascertained a limited benefit from the use of duloxetine, a selective serotonin and noradrenalin reuptake inhibitor, on the established CIPN [3].

We have demonstrated that high mobility group box 1 (HMGB1), one of the damage-associated molecular patterns (DAMPs), plays a role in the development and maintenance of CIPN in rodents treated with paclitaxel, vincristine, or oxaliplatin [2,4,5,6,7]. HMGB1, a nuclear protein, once released to the extracellular space, activates the receptor for advanced glycation end-products (RAGE) and Toll-like receptors (TLRs), including TLR4 and TLR5, and accelerates CXCL12/CXCR4 signals, thereby promoting inflammation and pain sensations [7,8,9,10]. Thus, HMGB1 and its membrane receptors are now considered promising targets for the prevention or treatment of CIPN. Most interestingly, thrombomodulin (TM), an endothelial membrane protein, accelerates thrombin-dependent degradation of extracellular HMGB1, an effect being reproduced by recombinant human soluble TM (TMα) that is composed of TM’s extracellular domains and clinically used as a therapeutic agent for disseminated intravascular coagulation (DIC) in Japan [11]. We have shown that TMα as well as an anti-HMGB1-neutralizing antibody prevents CIPN in rodents treated with paclitaxel, vincristine, or oxaliplatin [4,5,6]. Of importance is that the clinical efficacy of TMα in preventing CIPN has been demonstrated in colorectal cancer patients undergoing oxaliplatin-based chemotherapy [12]. It is noteworthy that the thrombin-dependent anti-CIPN effect of TMα is cancelled by anticoagulants and that repeated treatment with anticoagulants itself increases plasma HMGB1 levels and aggravates CIPN [2,6,7]. Thus, the endogenous TM/thrombin system appears to function to degrade HMGB1 in mice treated with oxaliplatin, thereby suppressing CIPN development. 

The origins of endogenous HMGB1 responsible for CIPN development appear to differ depending on chemotherapeutics employed, e.g., CIPN following treatment with paclitaxel and oxaliplatin involves HMGB1 derived mainly from macrophages and non-macrophage cells, respectively [2,5,7]. In macrophages, paclitaxel causes cytoplasmic translocation and extracellular release of nuclear HMGB1 following the upregulation of histone acetyltransferases (HATs) through reactive oxygen species (ROS) accumulation and the activation of p38 MAP kinase (p38MAPK) and NF-κB signals, but independently of JAK/STAT, calcium/calmodulin-dependent protein kinase (CaMK) and MAP kinases [2,5,7] that are involved in lipopolysaccharide (LPS)-induced HMGB1 release from macrophages [13,14,15].

Bortezomib, a proteasome inhibitor that is used to treat multiple myeloma, also often causes CIPN. The detailed mechanisms of CIPN caused by bortezomib have yet to be clarified, although there is evidence for the involvement of ROS generation and T-type calcium channels, particularly of Ca_v_3.2 form [16,17,18]. In the present study, we thus examined the possible involvement of macrophage-derived HMGB1 in a mouse model of CIPN caused by bortezomib. We then analyzed the molecular mechanisms for bortezomib-induced HMGB1 release from macrophages. Here, we show, for the first time to our knowledge, that bortezomib directly stimulates HMGB1 release from macrophages in a manner dependent on caspase, which is different from the mechanism for paclitaxel-induced HMGB1 release form macrophages, contributing to the development of CIPN in mice treated with bortezomib.

## 2. Materials and Methods

### 2.1. Animals

Male ddY mice (4–5 weeks old) were purchased from Kiwa Laboratory Animals Co., Ltd. (Wakayama, Japan) and bred in a room maintained at about 24 ℃ under a 12-h day/night cycle with free access to food and water. All protocols for animal experiments were approved by the Committee for the Care and Use of Laboratory Animals at Kindai University (KAPS-27-001 approved on 1 April 2015, and KAPS-2020-018 approved on 1 April 2020) and were accordance with the NIH guidelines (Guide for Care and Use of Animals, NIH Publication 86-23).

### 2.2. Major Chemicals

Bortezomib was purchased from LC Laboratories (Woburn, MA, USA). Anti-HMGB1-neutralizing antibody and control IgG were made in house, the specificity of the antibody being reported elsewhere [19]. Recombinant human soluble thrombomodulin (thrombomodulin alfa, TMα) was a gift from Asahi Kasei Pharma Corporation (Tokyo, Japan). Argatroban, AMD3100, TH1020, minocycline, ethyl pyruvate, U0126, STO-609, pyrrolidine dithiocarbamate (PDTC), and N-acetyl-L-cysteine (NAC) were purchased from Sigma-Aldrich (St. Louis, MO, USA). FPS-ZM1, SB203580, and SP600125 were obtained from Calbiochem (Temecula, CA, USA). TAK-242, MG132, and pyridine 6 were purchased from Cayman Chemical Company (Ann Arbor, MI, USA), Merck-Millipore (Darmstadt, Germany), and FUJIFILM Wako Pure Chemical Corporation (Osaka, Japan), respectively. Liposomal clodronate (Clophosome-A^®^) and the control liposome were obtained from FormuMax Scientific, Inc. (Sunnyvale, CA, USA). Z-VAD-FMK and Z-IETD-FMK were purchased from R&D Systems, Inc. (Minneapolis, MN, USA). Bortezomib, TAK-242, TH1020, and Z-VAD-FMK were dissolved in dimethyl sulfoxide (DMSO) and diluted with saline for in vivo study. The anti-HMGB1-neutralizing antibody and IgG were dissolved in 0.01 M phosphate-buffered saline (PBS), and TMα was in saline containing 0.002% Tween 80. FPS-ZM1 was dissolved in saline containing 0.5% DMSO and 10% Tween 80. Argatroban, AMD3100, minocycline, ethyl pyruvate, and PDTC were dissolved in saline. MG132, SB203580, SP600125, pyridine 6, STO-609, Z-IETD-FMK were dissolved in DMSO.

### 2.3. Assessment of Mechanical Nociceptive Threshold and Creation of a Mouse Model of CIPN Caused by Bortezomib

The mouse was placed in a transparent plastic box (10 × 10 × 10 cm) on a risen wire mesh floor. After acclimatization to the environment for 1 h, the surface of mid-planter in hindpaw was stimulated with von Frey filaments of distinct strengths (0.008, 0.02, 0.04, 0.07, 0.16, 0.4, 0.6, or 1.0 g). The 50% nociceptive threshold was calculated using an up-down method [20]. Indeed, the stimulation with filaments was started with a middle intensity of 0.07 g. In the absence of a paw withdrawal, the stimulation was performed by one stronger filament and repeated until the observation of positive response. If a paw withdrawal was observed, one weaker filament was used for next stimulation. The stimulation was performed 5 times, including the first positive response. The pattern for the positive or negative responses was recorded and the 50% response threshold was calculated using the following formula:

50% g threshold = [10 ^(Xf + kδ)^]/10,000

Xf = value in log units of the final von Frey filament; k = tabular value for the pattern of positive/negative responses; δ = mean difference in log units between stimuli. Measurements of nociceptive threshold in critical experiments were performed in a blinded fashion. For the induction of CIPN, mice received i.p. bortezomib at 0.1–0.4 mg/kg on days 0, 2, 5, 7, 9, and 12, as described previously [18,21].

### 2.4. Drug Administration Schedules

In experiments to test the preventive effects, the anti-HMGB1-neutralizing antibody at 1 mg/kg, control IgG at 1 mg/kg, TMα at 1 or 3 mg/kg, FPS-ZM1 at 1 mg/kg, TAK-242 at 3 mg/kg, AMD3100 at 8 mg/kg, TH1020 at 1 mg/kg, ethyl pyruvate at 80 mg/kg, or Z-VAD-FMK at 1 mg/kg was administered i.p. 30 min before each administration of bortezomib at 0.4 mg/kg on days 0, 2, 5, 7, 9, and 12, i.e., 6 times in total. Minocycline was administered i.p. 24 h before the first dose of bortezomib and 30 min before each dose of bortezomib on days 0, 2, 5, 7, 9, and 12, i.e., 7 times in total. To examine the therapeutic effects, the anti-HMGB1-neutralizing antibody, IgG, TMα, FPS-ZM1, TAK-242, AMD3100, TH1020, minocycline, or ethyl pyruvate was administered i.p. on day 14 after the onset of bortezomib treatment. To examine the effect of argatroban on the TMα-induced prevention of CIPN following treatment with bortezomib at 0.4 mg/kg, argatroban at 10 mg/kg was administered i.p. 30 min before each dose of TMα at 3 mg/kg, on days 0, 2, 5, 7, 9, and 12, i.e., 6 times in total. To test the effect of argatroban on the development of CIPN itself, argatroban at 10 mg/kg was administered i.p. once a day on days 0–13 in mice treated with bortezomib at a low dose, 0.1 mg/kg, on days 0, 2, 5, 7, 9, and 12.

### 2.5. Determination of Protein Levels of HMGB1 and Its Targeted Receptors

On day 14 after the onset of bortezomib treatment, mice were anesthetized with i.p. pentobarbital at 10 mg/kg following i.p. administration of a mixture of midazolam at 4 mg/kg and medetomidine at 0.3 mg/kg. After blood was collected from the abdominal aorta, the mouse was sacrificed by exsanguination. As described previously [22], after the skin and muscles on the back side were incised to expose the spine (chest to lumbar region), the spinal canal was cut open, and the bilateral dorsal root ganglia (DRG) at L4-L6 spinal levels were excised under microscope observation. Thereafter, the bilateral sciatic nerves were excised from the left and right thighs. Plasma HMGB1 levels were analyzed using an ELISA kit (Shino-Test Corporation, Tokyo, Japan). Protein levels of HMGB1, RAGE, TLR4, and CXCR4 in the DRG and sciatic nerve were measured by Western blotting, as reported previously [23]. Primary antibodies were: an anti-HMGB1 rabbit polyclonal antibody (Abcam, Cambridge, UK) (1: 5000 dilution), anti-RAGE rabbit polyclonal antibody (Abcam) (1:1000 dilution), anti-TLR4 rabbit polyclonal antibody (Santa Cruz Biotechnology, Inc., Santa Cruz, CA, USA) (1:10000 dilution), anti-CXCR4 rabbit polyclonal antibody (Novus Biologicals, Littleton, CO, USA) (1:1000 dilution), and anti-glyceraldehyde 3-phosphate dehydrogenase (GAPDH) rabbit polyclonal antibody (Santa Cruz Biotechnology, Inc.) (1:5000 dilution). A horseradish peroxidase (HRP)-conjugated anti-rabbit IgG goat antibody (Cell Signaling Technology, Beverly, MA, USA) (1:5000 dilution) was used as a secondary antibody. The immunoreactive proteins were visualized with a chemiluminescence detection reagent (Chemi-Lumi One Super, Nacalai Tesque, Inc., Kyoto, Japan), and the bands were detected by Image Quant 400 (GE Healthcare, Little Chalfont, Buckinghamshire, UK) and quantified using densitometric software (ImageJ downloaded from https://imagej.nih.gov/ij/download.html (1 August 2019).

### 2.6. Depletion of Macrophages in Mice

Liposomal clodronate at 1.05 mg/mouse was administered i.p. on days 5 and 7 of bortezomib treatment, and nociceptive threshold was measured daily until day 8. To confirm the successful macrophage depletion, the spleen was isolated from the mice that were sacrificed by cervical dislocation following the measurement of nociceptive threshold on day 8 after the onset of bortezomib treatment and subjected to detection of macrophages by flow cytometry using FITC anti-mouse F4/80 (1:100; Biolegend, Inc., San Diego, CA, USA) and PE anti-mouse/human CD11b (1:200; Biolegend, Inc.), as described previously [23]. To check the effect of macrophage depletion on the established CIPN after bortezomib treatment, the mice received a single i.p. administration of liposomal clodronate at 1.05 mg/mouse on day 13 after the onset of i.p. bortezomib.

### 2.7. Analysis of Macrophage Accumulation in the Sciatic Nerve and DRG by Immunofluorometry

Under anesthesia with i.p. pentobarbital at 10 mg/kg following i.p. administration of a mixture of midazolam at 4 mg/kg and medetomidine at 0.3 mg/kg, mice were transcardially perfused with ice-cold saline in a volume of 20 mL and then with 4% paraformaldehyde (PFA) in 0.1 M phosphate buffer in a volume of 50 mL. The sciatic nerve and L4-L6 DRG were removed and subjected to immunohistochemical detection of macrophages, as described previously [5]. Macrophages were visualized by immunofluorometric analysis, using a rat anti-mouse F4/80 monoclonal antibody (Bio-rad, Hercules, CA, USA) (1:1000 dilution), Cy3-conjgated goat anti-rat IgG antibody (Thermo Fisher Scientific, Carlsbad, CA, USA) (1:400 dilution), and 4,6-diamidino-2-phenylindole dihydrochloride (DAPI) (Sigma-Aldrich) (1:2000 dilution), as reported previously [5]. The F4/80-positive cells were recognized by confocal laser fluorescence microscopy (FV10C-O, Olympus, Tokyo, Japan). The number of F4/80-positive cells were counted on each visual field.

### 2.8. Cell Culture

Mouse macrophage-like RAW264.7 cells were cultured in RPMI1640 medium (Nacalai Tesque, Inc., Kyoto, Japan) containing 10% fetal calf serum (FCS) (Nichirei Bioscience, Tokyo, Japan), 100 U/mL penicillin (Nacalai Tesque, Inc.), 100 µg/mL streptomycin (Nacalai Tesque, Inc.) in a 5% CO_2_ incubator at 37 °C.

### 2.9. Determination of Expression, Release, and Cytoplasmic Translocation of Nuclear HMGB1 and Protein Levels of Cleaved Caspase-8 in RAW264.7 Cells

RAW264.7 cells were seeded and cultured in a 6-well plate (12 × 10^4^ cell/well) or 60-mm diameter culture dish (36 × 10^4^ cell/dish) filled with 10% FCS-containing culture medium for 24 h. After additional 24-h incubation in 1% FCS-containing medium in a volume of 1 mL, the cells stimulated with bortezomib at 0.1–100 nM for 9–24 h or MG132 at 0.1–0.3 μM for 12 h [24,25]. Then, exactly 0.5 mL of the supernatant medium was collected and centrifugated. After the assessment of protein content by the Bradford method, HMGB1 levels in the supernatant were determined using an ELISA kit (Fuso Pharmaceutical Industries, Ltd., Osaka, Japan) or by Western blotting using the above-mentioned antibodies, as described elsewhere [5]. Protein levels of cleaved caspase-8 in the pellets were determined by Western blotting using an anti-mouse cleaved caspase-8 (Asp387) rabbit antibody (1:1000 dilution) that recognizes both p43 (pro-domain with p18) and p18, the cleaved products of caspase-8 when caspase-8 was cleaved at Asp387 (Cell Signaling Technology), together with the above-mentioned secondary antibody.

For the immunostaining experiments, RAW264.7 cells (3 × 10^4^ cells/well) were seeded on cover glass in a 24-well plate filled with 10% FCS-containing culture medium and stimulated as described above. The cells, after stimulated with bortezomib at 10 nM for 9 h, were fixed with 4% PFA in PBS. The subcellular localization of HMGB1 was detected by immunofluorescence staining using an anti-HMGB1 rabbit polyclonal antibody (Shino-Test Corporation) (1:500 dilution), TRITC-conjugated anti-rabbit IgG (Sigma-Aldrich) (1:400 dilution), and DAPI (1:200 dilution), as described previously [5]. Fluorescence images were visualized using a confocal laser fluorescence microscopy (FV10C-O, Olympus). Translocation of HMGB1 from the nucleus to cytoplasm were calculated as the ratio of nuclear HMGB1 to cytoplasmic HMGB1 using FV10-ASW software (Olympus).

### 2.10. Detection of Nuclear Condensation or Fragmentation by Hoechst Staining

RAW264.7 cells were seeded and cultured in a 6-well plate (12 × 10^4^ cell/well) containing 10% FCS-containing culture medium for 24 h. After additional 24-h incubation in 1% FCS-containing medium, the cellsstimulated for 12 h with bortezomib at 10 nM, which was a concentration capable of releasing HMGB1 in the preliminary experiments. The cells were collected and fixed with 1% glutaraldehyde for 30 min at room temperature. After washing with PBS, the cells were stained with Hoechst 33342 (Sigma-Aldrich) at 1 mM for 15 min. The stained cells were placed on a slide glass and covered with a cover glass. The number of cells with nuclear condensation or fragmentation was counted under UV excitation light with an inverted fluorescence microscope (BX50, Olympus, Tokyo, Japan).

### 2.11. Statistics

Data are shown as mean ± S.E.M. Statistical significance for parametric data was analyzed by Student’s *t*-test for two-group data, and by ANOVA followed by Tukey’s post hoc test for multiple comparisons. In some experiments, two-way factorial ANOVA was also employed. For non-parametric data, Kruskal–Wallis H-test followed by the LSD test was used. A significant difference was set a *p* < 0.05.

## 3. Results

### 3.1. Involvement of HMGB1 in the CIPN Caused by Bortezomib in Mice

**Figure 1:**
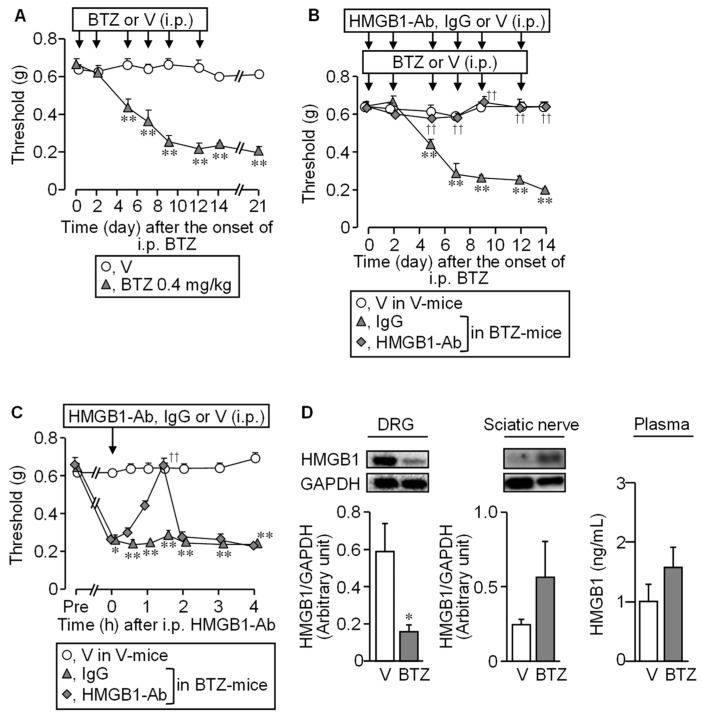
Involvement of HMGB1 in CIPN caused by bortezomib in mice. (**A**) The time course of nociceptive thresholds in mice that received repeated i.p. administration of bortezomib at 0.4 mg/kg or vehicle on days 0, 2, 5, 7, 9, and 12. (**B**,**C**) Preventive (**B**) and therapeutic (**C**) effects of an anti-HMGB1-neutralizing antibody on the bortezomib-induced mechanical allodynia in mice. The mice received repeated i.p. administration of an anti-HMGB1-neutralizing antibody at 1 mg/kg, IgG at 1 mg/kg or vehicle 30 min before each dose of bortezomib or vehicle (**B**) or single i.p. administration of each of them after the establishment of CIPN on day 14 (**C**). (**D**) The protein levels of HMGB1 in the dorsal root ganglion (DRG), sciatic nerve (Western blotting), or plasma (ELISA) on day 14 after the onset of bortezomib treatment. Typical photographs of blotting are shown on the top above columns Data show the mean with S.E.M for 5 (**A**–**C**) or 6 (**D**) mice. V, vehicle; BTZ, bortezomib; HMGB1-Ab, anti-HMGB1-neutralizing antibody. * *p* < 0.05, ** *p* < 0.01 vs. V (**A**,**D**) or V in V-treated mice (**B**,**C**). ^††^ *p* < 0.01 vs. IgG in BTZ-treated mice (B,C).

The repeated i.p. administration of bortezomib at 0.4 mg/kg to mice gradually lowered the mechanical nociceptive threshold from day 5, which reached a bottom on days 9 to 12. This mechanical allodynia lasted to day 21 and later (Figure 1A).

An anti-HMGB1-neutralizing antibody, when given i.p. at 1 mg/kg before each dose of bortezomib, six times in total, completely prevented the development of the CIPN following bortezomib treatment (Figure 1B). Interestingly, the anti-HMGB1-neutraling antibody, when administered on day 14 after the onset of bortezomib treatment, transiently elevated the nociceptive threshold lowered by bortezomib in the mice, an effect quickly disappearing thereafter (Figure 1C). Thus, HMGB1 inactivation after the establishment of CIPN could not reverse the progression of CIPN in bortezomib-treated mice, as shown in rodent models for CIPN caused by paclitaxel and oxaliplatin [4,6]. Protein levels of HMGB1 tended to increase in the sciatic nerves and plasma, although it significantly decreased in the DRG (Figure 1D), in agreement with results in rats with CIPN caused by paclitaxel or vincristine [4].

### 3.2. TM/TMα Prevents and Reverses the CIPN Caused by Bortezomib in a Thrombin-Dependent Manner in Mice

**Figure 2:**
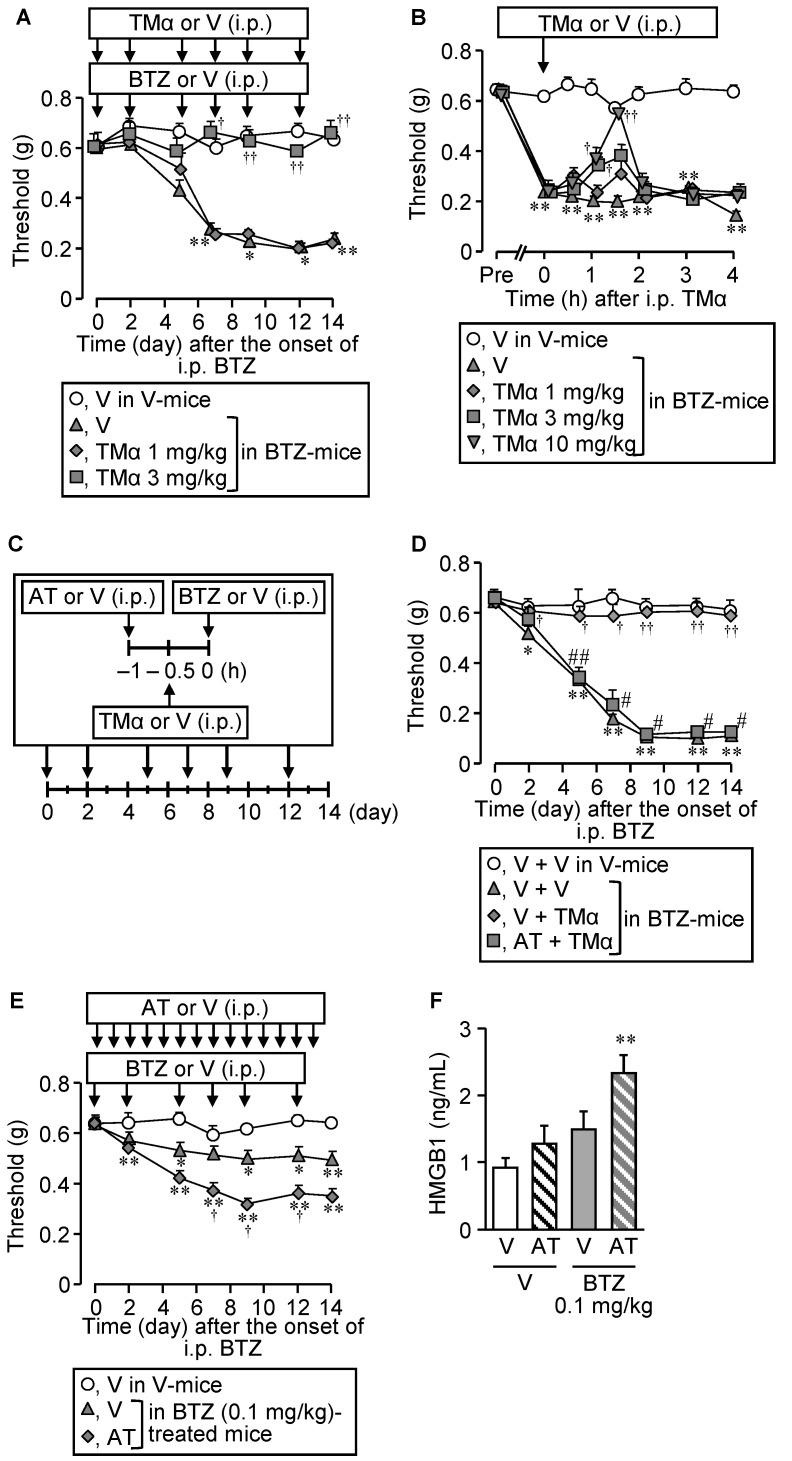
Prevention and reversal of the CIPN caused by bortezomib by thrombomodulin alfa, and the role of endogenous thrombin. (**A**,**B**) Preventive (**A**) or therapeutic (**B**) effects of thrombomodulin alfa on the CIPN caused by bortezomib in mice. Bortezomib at 0.4 mg/kg or vehicle was administered i.p. on days 0, 2, 5, 7, 9, and 12. The mice received repeated i.p. administration of thrombomodulin alfa at 1 or 3 mg/kg, 30 min before each dose of bortezomib (**A**), or a single i.p. administration of thrombomodulin alfa at 1, 3, or 10 mg/kg after the establishment of CIPN on day 14 (**B**). (**C**,**D**) Cancellation of the anti-CIPN effect of TMα by argatroban, a thrombin inhibitor. The mice received repeated i.p. administration of argatroban at 10 mg/kg, 30 min before each administration of thrombomodulin alfa at 10 mg/kg (60 min before each administration of bortezomib at 0.4 mg/kg) (**C**). (**E**,**F**) Long-term inhibition of endogenous thrombin by argatroban promotes the CIPN (**E**) and increased plasma HMGB1 levels (**F**) in mice treated with bortezomib at 0.1 mg/kg, a subeffective dose. The mice received repeated i.p. administration of argatroban at 10 mg/kg once a day on days 0–13 after the onset of treatment with bortezomib. Plasma HMGB1 levels were determined after the measurement of nociceptive threshold on day 14 (**F**). Data show the mean with S.E.M for 4–6 (**A**), 5–6 (**B**), 5 (**D**), 9–13 (**E**), or 9–11 (**F**) mice. V, vehicle; BTZ, bortezomib; TMα, thrombomodulin alfa; AT, argatroban. * *p* < 0.05, ** *p* < 0.01 vs. V in V-treated mice (**A**,**B**,**E**,**F**) or V + V in V-treated mice (**D**). ^†^ *p* < 0.05, ^††^ *p* < 0.01 vs. V in BTZ-treated mice (**A**,**B**,**E**,**F**) or V + V in BTZ-treated mice (**D**). ^#^ *p* < 0.05, ^##^ *p* < 0.01 vs. V + TMα in BTZ-treated mice (**D**).

As did the anti-HMGB1-neutralizing antibody (see Figure 1B,C), TMα, capable of promoting thrombin-dependent degradation of HMGB1, when given i.p. repeatedly at 3 mg/kg, completely prevented the development of the CIPN following repeated treatment with bortezomib at 0.4 mg/kg (Figure 2A).

It is to note that repeated treatment with TMα itself did not alter nociceptive threshold in vehicle-treated mice (Supplementary Figure S1). TMα also reversed the established CIPN when administered i.p. at 3 or 10 mg/kg once on day 14 after the onset of bortezomib treatment (Figure 2B). Argatroban, a parenteral direct thrombin inhibitor, when administered i.p. at 10 mg/kg, 30 min before each administration of TMα at 3 mg/kg, 6 times in total (Figure 2C), abolished the anti-CIPN effect of TMα in the mice treated with bortezomib at 0.4 mg/kg (Figure 2C,D). Most interestingly, in the mice that received repeated i.p. administration of bortezomib at a low dose, 0.1 mg/kg, repeated i.p. administration of argatroban at 10 mg/kg significantly facilitated the development of allodynia (Figure 2E) and elevated the increased plasma HMGB1 levels (Figure 2F).

### 3.3. HMGB1-Targeted Receptors Involved in the CIPN Caused by Bortezomib in Mice

**Figure 3:**
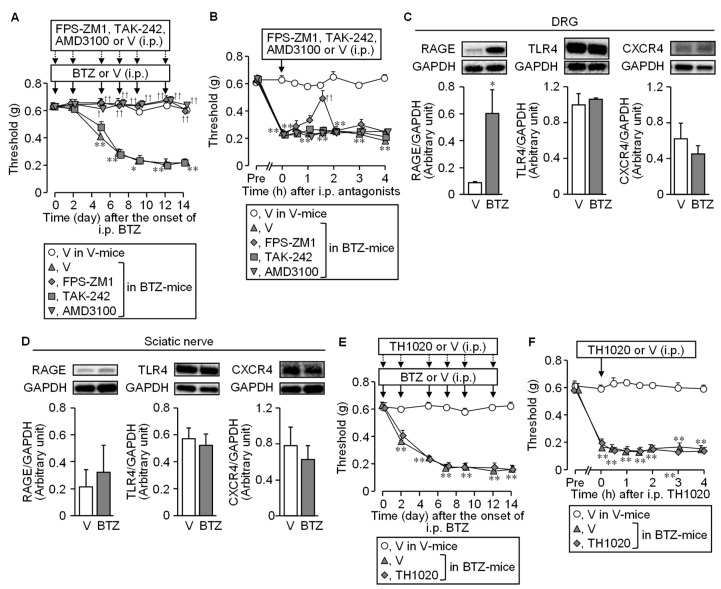
Involvement of HMGB1-targeted receptors in the CIPN caused by bortezomib. Bortezomib at 0.4 mg/kg or vehicle was administered i.p. on days 0, 2, 5, 7, 9, and 12. (**A**,**B**) Preventive (**A**) or therapeutic (**B**) effect of FPS-ZM1, TAK-242, and AMD3100, antagonists of RAGE, TLR4, and CXCR4, respectively. The mice received repeated i.p. administration of FPS-ZM1 at 1 mg/kg, TAK-242 at 3 mg/kg, AMD3100 at 8 mg/kg, or vehicle, 30 min before each dose of bortezomib (**A**), or single i.p. administration of each of them on day 14 (**B**). (**C**,**D**) Protein levels of RAGE, TLR4, and CXCR4 in the dorsal root ganglion (**C**) and sciatic nerves (**D**) on day 14 after the onset of bortezomib treatment. (**E**,**F**) Lack of preventive (**E**) and therapeutic (**F**) effects of TH1020, a TLR5 antagonist. The mice received repeated (**E**) or single (**F**) i.p. administration of TH1020 at 1 mg/kg or vehicle, in mice treated with bortezomib according to the above-mentioned schedules. Data show the mean with S.E.M for 5–6 (**A**,**B**,**F**), 5–7 (**C**), 6–7 (**D**), or 6 (**E**) mice. V, vehicle; BTZ, bortezomib; DRG, dorsal root ganglion; * *p* < 0.05, ** *p* < 0.01 vs. V (**C**) or V in V-treated mice (**A**,**B**,**E**,**F**). ^†^ *p* < 0.05, ^††^ *p* < 0.01 vs. V in BTZ-treated mice.

We next tested whether the CIPN caused by bortezomib involves the HMGB1-targeted membrane receptors, such as RAGE, TLR4, and CXCR4, known to participate in the CIPN caused by paclitaxel or oxaliplatin [4,5,6]. FPS-ZM1 at 1 mg/kg, a RAGE antagonist, and AMD3100 at 8 mg/kg, a CXCR4 antagonist, when administered i.p. 30 min before each administration of bortezomib at 0.4 mg/kg, 6 times in total, completely prevented the development of CIPN following repeated bortezomib treatment, whereas TAK-242, a TLR4 antagonist, administered at 3 mg/kg in the same schedule, did not exhibit such an anti-CIPN effect (Figure 3A).

It is to note that repeated treatment with FPS-ZM1 itself did not alter nociceptive threshold in vehicle-treated mice (Supplementary Figure S1). Most interestingly, FPS-ZM1 but not AMD3100 or TAK-242, given i.p. once on day 14 after the onset of bortezomib, transiently reversed the established CIPN in the mice treated repeatedly with bortezomib at 0.4 mg/kg (Figure 3B). These behavioral results are apparently in agreement with the overexpression of RAGE, but not TLR4 or CXCR4, in the DRG observed on day 14 after the onset of bortezomib treatment (Figure 3C), although RAGE was not upregulated in the sciatic nerves of the bortezomib-treated mice (Figure 3D). There is a report showing the involvement of TLR5 in the allodynia induced by exogenously applied HMGB1 in mice [26]. However, TH1020, a TLR5 antagonist, when administered i.p. at 1 mg/kg repeatedly and once in the above-described manner, neither prevented nor reversed the CIPN in bortezomib-treated mice, respectively (Figure 3E,F).

### 3.4. Involvement of Macrophages in the Developmental Stage of CIPN following Bortezomib Treatment in Mice

**Figure 4:**
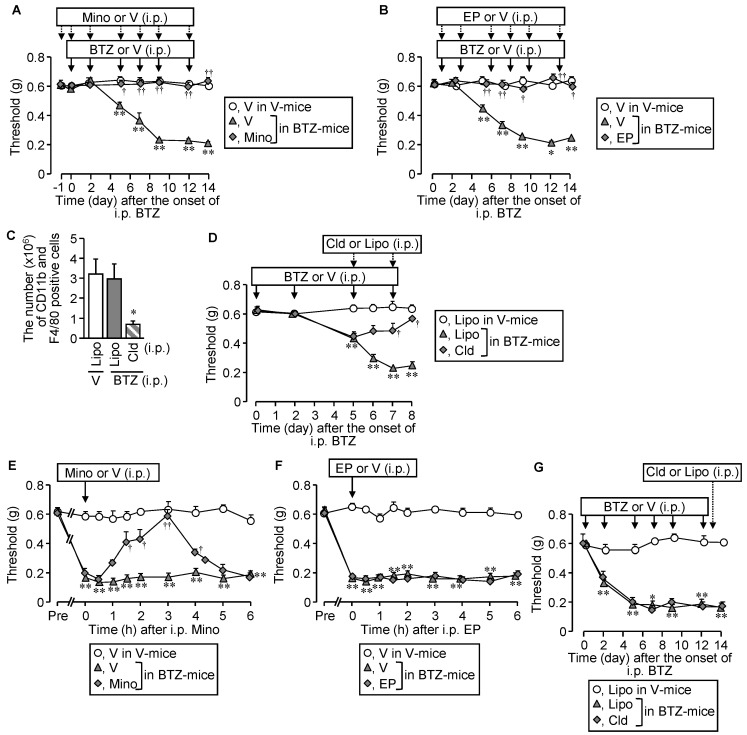
Effect of macrophage inhibition or depletion on the development of CIPN in mice treated with bortezomib. Bortezomib at 0.4 mg/kg or vehicle was administered i.p. on day 0, 2, 5, 7, 9, and 12. (**A**,**B**) Preventive effects of minocycline, a macrophage/microglia inhibitor, and ethyl pyruvate, known to inhibit HMGB1 release from macrophages. Minocycline at 30 mg/kg was administered i.p. once 24 h before the onset of bortezomib treatment and repeatedly 30 min before each dose of bortezomib (**A**). Ethyl pyruvate at 80 mg/kg was administered i.p. 1 h before each dose of bortezomib (**B**). (**C**,**D**) Effect of macrophage depletion with liposomal clodronate on the development of CIPN caused by bortezomib. The mice received two i.p. administrations of liposomal clodronate at 1.05 mg/mouse or the control liposome on days 5 and 7 after the onset of bortezomib treatment, and successful macrophage depletion in the isolated spleen was confirmed by counting CD11b- and F4/80-positive macrophages in flow cytometry (**C**). (**E**–**G**) Effects of minocycline, ethyl pyruvate, and liposomal clodronate on the established CIPN in mice treated with bortezomib. The mice received a single i.p. administration of minocycline or ethyl pyruvate on day 14 and liposomal clodronate or the control liposome on day 13 after the onset of bortezomib treatment. V, vehicle; BTZ, bortezomib; Mino, minocycline; EP, ethyl pyruvate; Cld, liposomal clodronate; Lipo, control liposome. Data show the mean with S.E.M for 5–6 (**A**,**B**,**D**,**E**,**F**), 7–9 (**G**), or 5 (**H**) mice. * *p* < 0.05, ** *p* < 0.01 vs. V in V-treated mice (**A**,**B**,**E**–**G**) or Lipo in V-treated mice (**C**,**D**,**G**). ^†^ *p* < 0.05, ^††^ *p* < 0.01 vs. V in BTZ-treated mice (**A**,**B**,**E**) or Lipo in BTZ-treated mice (**D**).

Given evidence that the origins of endogenous HMGB1 responsible for CIPN development appear to differ depending on chemotherapeutics employed [2,5,6,7], we asked if different inhibitors or a depletor of macrophages could affect the CIPN caused by bortezomib. Repeated i.p. administration of minocycline at 30 mg/kg, a macrophage/microglia inhibitor, or ethyl pyruvate at 80 mg/kg, known to inhibit HMGB1 release from macrophages, blocked the CIPN development following bortezomib treatment (Figure 4A,B).

Liposomal clodronate, a macrophage depletor, when administered i.p. twice at 1.05 mg/mouse on days 5 and 7, dramatically decreased the number of macrophages in the spleen (Figure 4C), and significantly reduced the CIPN development following bortezomib treatment (Figure 4D). A single administration of minocycline at the same dose, known to inhibit not only peripheral macrophages but also spinal microglia, significantly reversed the established CIPN in bortezomib-treated mice (Figure 4E), although neither ethyl pyruvate nor liposomal clodronate affected the established CIPN (Figure 4F,G).

**Figure 5:**
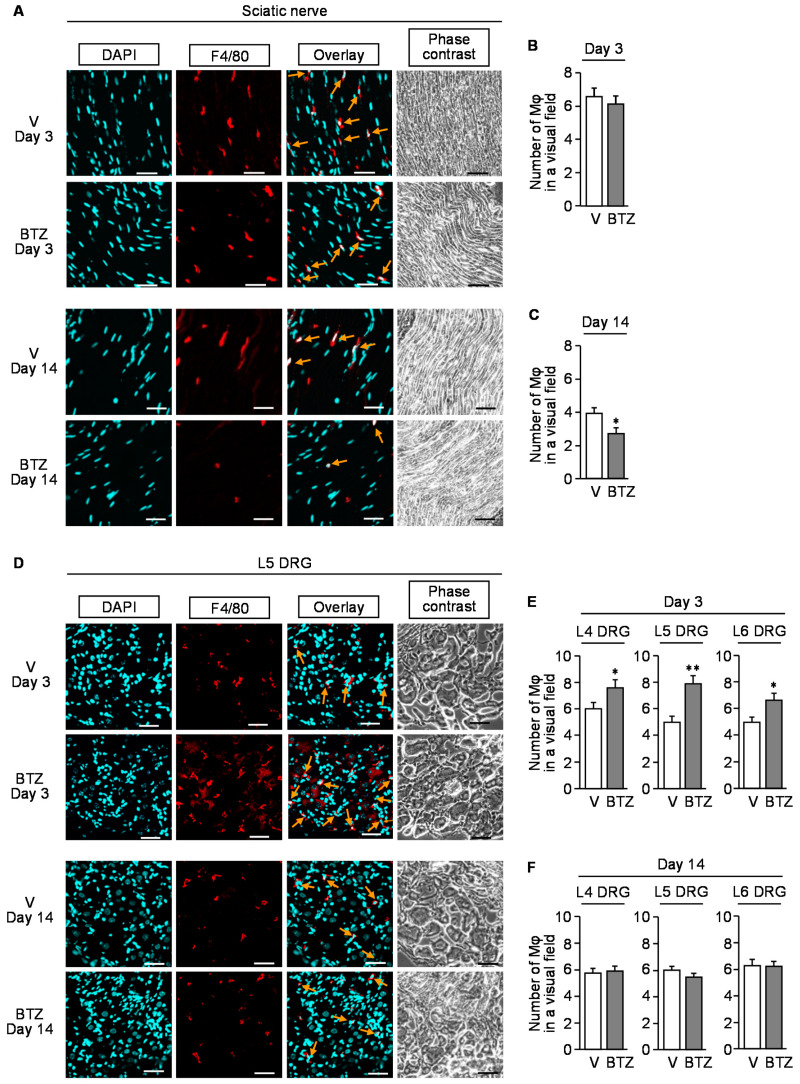
Immunofluorescence detection of macrophages in the sciatic nerve or dorsal root ganglion of the mice with CIPN caused by bortezomib. Bortezomib at 0.4 mg/kg or vehicle was administered i.p. on days 0, 2, 5, 7, 9, and 12. The sciatic nerves (**A**–**C**) and dorsal root ganglion (**D**–**F**) were isolated from the mice on day 3 or 14 after the onset of bortezomib treatment. (**A**,**D**) Typical microphotographs for the immunofluorescence staining of F4/80-positive macrophages (arrows) in the sciatic nerve (**A**) and L5 dorsal root ganglion (**D**). Blue, DAPI (nucleus); red, F4/80 (macrophage); scale bar, 50 μm. (**B**,**C**,**E**,**F**) The number of F4/80-positive cells in a visual field of the sciatic nerve (**B**,**C**) and L4–L6 dorsal root ganglion (**E**,**F**) excised on day 3 (**B**,**E**) and day 14 (**C**,**F**). Mφ, macrophage; V, vehicle; DRG, dorsal root ganglion; BTZ, bortezomib. Data show the mean with S.E.M for 20 visual fields from 5 mice. * *p* < 0.05, ** *p* < 0.01 vs. V.

Immunofluorescent analyses showed that repeated treatment with bortezomib caused macrophage accumulation in DRG at L4–L6 spinal levels but not in the sciatic nerve on day 3 after the onset of bortezomib treatment (Figure 5A,B,D,E), while no such increase in the number of macrophages was detected in the sciatic nerve or DRG on day 14 after the onset of bortezomib treatment (Figure 5A,C,D,F). It is noteworthy that there were significant (*p* < 0.001) differences in the number of macrophages between days 3 and 14 when analyzed by two-way ANOVA, which might the reflect non-specific effect of repeated i.p. injections or variations on measurement because of unexpected biases.

### 3.5. Bortezomib and MG132, Proteasome Inhibitors, Evoke HMGB1 Release from Macrophage-Like RAW264.7 Cells

**Figure 6:**
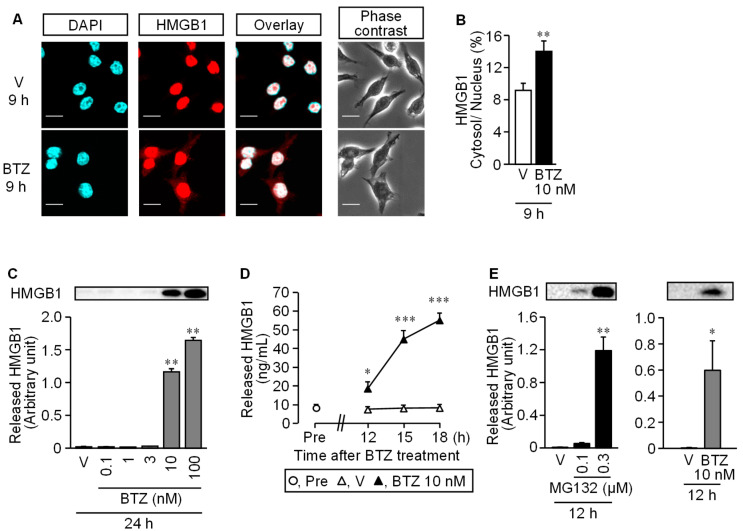
Cytoplasmic translocation and extracellular release of nuclear HMGB1 in RAW264.7 macrophages stimulated with bortezomib. (**A**,**B**) Translocation of HMGB1 from the nucleus to the cytoplasm in RAW264.7 cells. The cells were stimulated with bortezomib at 10 nM for 9 h, and HMGB1 was detected by immunofluorescence staining. Blue, DAPI (nucleus); red, HMGB1; Scale bar, 50 μm (**A**). (**B**) Proportion (%) of fluorescence signal of HMGB1 in the cytoplasm to that in the nucleus. (**C**) Extracellular release of HMGB1 from RAW264.7 cells stimulated with bortezomib at 0.1–100 nM for 24 h, as assessed by Western blotting. Typical photographs are shown on the top above columns. (**D**) Time-dependent HMGB1 release from RAW264.7 cells stimulated with bortezomib at 10 nM for 12, 15, or 18 h, as assessed by ELISA. (**E**) MG132, another well-known proteasome inhibitor, as well as bortezomib causes HMGB1 release from RAW264.7 cells. The cells were stimulated with MG132 at 0.1 or 0.3 µM or bortezomib at 10 nM for 12 h, and the extracellular HMGB1 was determined by Western blotting. Typical photographs are shown on the top above columns. Data show the mean with S.E.M for 5 different experiments (**B**,**C**,**D**,**E**). V, vehicle; BTZ, bortezomib. * *p* < 0.05, ** *p* < 0.01, *** *p* < 0.001 vs. V.

In mouse macrophage-like RAW264.7 cells, stimulation with bortezomib at 10 and/or 100 nM caused the cytoplasmic translocation of nuclear HMGB1 at 9 h (Figure 6A,B) and extracellular release of HMGB1 at 24 h, as assessed by Western blotting (Figure 6C).

The time-course experiments employing an ELISA kit for HMGB1 measurement showed that extracellular HMGB1 concentrations gradually increased during the time period from 12 to18 h after the onset of stimulation with 10 nM bortezomib (Figure 6D). MG132, a well-known proteasome inhibitor, at 0.3 μM, like bortezomib at 10 nM, caused HMGB1 release in 12 h (Figure 6E).

### 3.6. Involvement of Caspase in Bortezomib-Induced HMGB1 Release from RAW264.7 Macrophages

**Figure 7:**
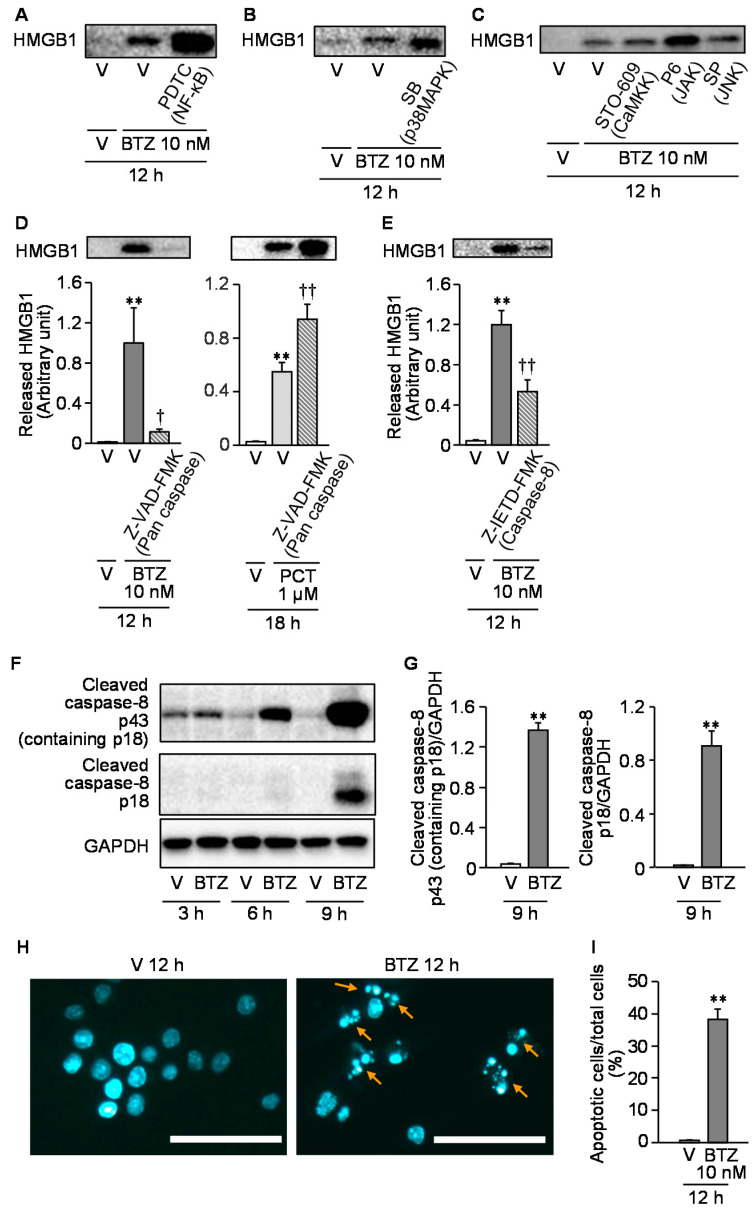
Analysis of cell signals involved in the bortezomib-induced HMGB1 release from RAW264.7 macrophages. (**A**-**C**) Lack of effects of pyrrolidine dithiocarbamate (**A**), SB203580 (**B**), STO-609, pyridone 6, and SP600125 (**C**), inhibitors of NF-κB, p38MAPK, CaMKK, JAK, and JNK, respectively, on the HMGB1 release from RAW264.7 cells stimulated with bortezomib. Pyrrolidine dithiocarbamate at 100 μM (**A**), SB203580 at 1 μM (**B**), STO-609 at 10 μg/mL, pyridone 6 at 30 nM, or SP600125 at 1 μM (**C**) was added 1 h before stimulation with bortezomib at 10 nM for 12 h, and the extracellular HMGB1 was detected by Western blotting. (**D**,**E**) Effects of Z-VAD-FMK, a pan-caspase inhibitor, and Z-IETD-FMK, a caspase 8-specific inhibitor, on the HMGB1 release from RAW264.7 cells stimulated with bortezomib. Z-VAD-FMK at 20 μM (**D**) or Z-IETD-FMK at 20 μM (**E**) was added 1 h before stimulation with bortezomib at 10 nM for 12 h (**D**,**E**) or paclitaxel at 1 μM for 18 h (**D**), and the extracellular HMGB1 was detected and quantified by Western blotting. Typical photographs are shown on the top above the columns of quantified data. Parentheses indicate molecular targets of each inhibitor (**A**–**E**). (**F**,**G**) Increase in protein levels of cleaved products of caspase-8, p43 (containing p18) and p18 following stimulation with bortezomib at 10 nM for 3–9 h (**F**) or for 9 h (**G**). The cleaved products were detected (**F**) and quantified (**G**) by Western blot analysis. (**H**,**I**) Detection of apoptotic cells after stimulation of bortezomib at 10 nM for 12 h. Nuclear fragmentation or condensation accompanying apoptosis (arrows) was detected by Hoechst 33342 staining (**H**), and the proportion (%) of apoptotic cells in a visual field was calculated (**I**). Scale bars indicate 50 μm. Data show the mean with S.E.M for 7 (**D** left), 5 (**D** right, **E**), or 8 (**G**) different experiments and for 20 visual fields from 5 different experiments (**I**). V, vehicle; BTZ, bortezomib; PDTC, pyrrolidine dithiocarbamate; SB, SB203580; P6, pyridone 6; SP, SP600125; PCT, paclitaxel. ** *p* < 0.01 vs. V (G, I) or V + V (**D**,**E**). ^†^ *p* < 0.05, ^††^ *p* < 0.01 vs. V + BTZ (**D** left, **E**) or V + PCT (**D** right).

We then tested effects of some inhibitors of cell signals on the bortezomib-induced HMGB1 release from RAW264.7 cells. Pyrrolidine dithiocarbamate (PDTC) and SB203580, inhibitors of NF-κB and p38MAPK, respectively, which abolished the paclitaxel-induced HMGB1 release from macrophages in our previous study [5], failed to reduce the bortezomib-induced HMGB1 release from RAW264.7 macrophages (Figure 7A,B).

STO-609, pyridone 6, and SP600125, inhibitors of the Ca^2+^/calmodulin-dependent protein kinase kinase (CaMKK), and JAK and JNK, respectively, which had been reported to block LPS-induced HMGB1 release from macrophages [13,14,15], did not significantly inhibit the bortezomib-induced HMGB1 release from RAW264.7 cells (Figure 7C). Given evidence for the relationship between apoptosis and HMGB1 release in macrophages [27,28], we next tested whether caspase inhibitors could inhibit the bortezomib-induced HMGB1 release from RAW264.7 cells. Z-VAD-FMK, a pan caspase inhibitor, at 20 μM completely inhibited the HMGB1 release from macrophages in response to bortezomib at 10 nM (Figure 7D). In contrast, surprisingly, Z-VAD-FMK rather promoted paclitaxel-induced HMGB1 release from macrophages (Figure 7D). We then found that Z-IETD-FMK, a caspase-8 inhibitor, at 20 μM significantly reduced the bortezomib-induced HMGB1 release from macrophages (Figure 7E) and that bortezomib treatment gradually increased protein levels of two cleaved products of caspase-8, p43 (containing p18) and p18, at 3–9 h in a time-dependent manner (Figure 7F,G). Actually, bortezomib at 10 nM caused apoptosis characterized by nuclear condensation or fragmentation in macrophages at 12 h (Figure 7H,I).

### 3.7. Systemic Administration of a Caspase Inhibitor Prevents the CIPN Caused by Bortezomib in Mice

**Figure 8:**
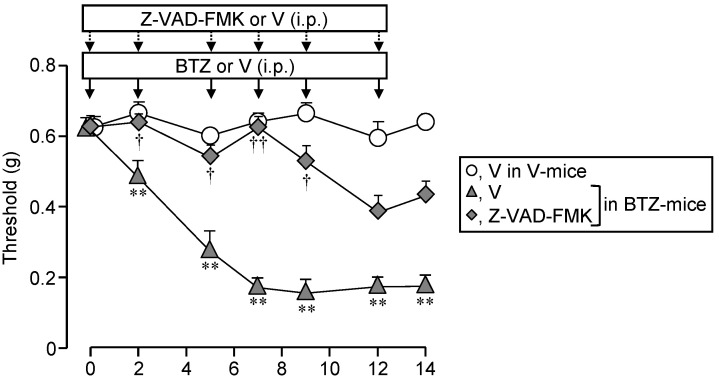
Repeated administration of Z-VAD-FMK, a pan-caspase inhibitor, completely prevents the development of CIPN following bortezomib treatment in mice. Bortezomib at 0.4 mg/kg or vehicle was administered i.p. on days 0, 2, 5, 7, 9, and 12. The mice received repeated i.p. administration of Z-VAD-FMK at 1 mg/kg, 30 min before each dose of BTZ. Data show the mean with S.E.M for 5 mice. V, vehicle; BTZ, bortezomib. ** *p* < 0.01 vs. V in V-treated mice. ^†^ *p* < 0.05, ^††^ *p* < 0.01 vs. V in BTZ-treated mice.

Finally, we then asked whether systemic administration of Z-VAD-FMK, the caspase inhibitor, could inhibit the CIPN in mice treated with bortezomib. Repeated i.p. administration of Z-VAD-FMK at 1 mg/kg significantly reduced the development of CIPN following repeated treatment with bortezomib at 0.4 mg/kg in the mice (Figure 8).

It is to note that repeated treatment with Z-VAD-FMK itself did not alter nociceptive threshold in vehicle-treated mice (Supplementary Figure S1).

## 4. Discussion

**Figure 9:**
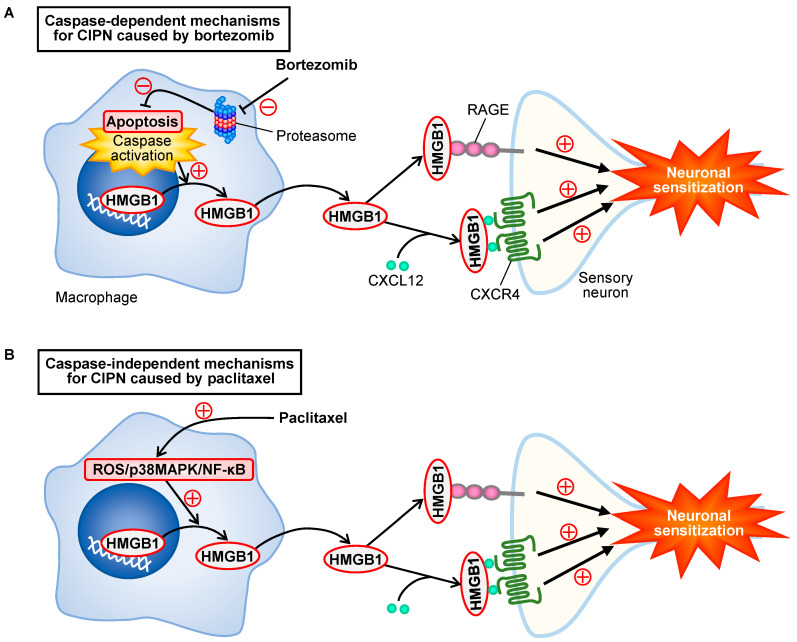
Scheme for bortezomib-induced caspase-dependent HMGB1 release from macrophages and CIPN development, in contrast to caspase-independent mechanisms for paclitaxel. (**A**) Inhibition of proteasome by bortezomib causes caspase-dependent apoptosis of macrophages followed by the release of HMGB1, which in turn causes neuronal sensitization via activation of RAGE and acceleration of CXCL12/CXCR4 signals, leading to CIPN. (**B**) Paclitaxel causes HMGB1 release from macrophages through activation of the ROS/p38MAPK/NF-κB pathway [5], independently of caspase (see Figure 7D), and the extracellular HMGB1 develops CIPN in a manner dependent on RAGE and CXCR4 [5], as shown in the CIPN caused by bortezomib.

The present study demonstrates the involvement of macrophage-derived HMGB1 in the development of CIPN in mice treated with bortezomib, in agreement with the mechanisms for the CIPN caused by paclitaxel [5]. Surprisingly, our findings from experiments employing RAW264.7 macrophages indicate that the bortezomib-induced HMGB1 release from macrophages involves caspase-dependent apoptosis but is independent of p38MAPK and NF-κB, which are involved in the paclitaxel-induced HMGB1 release from macrophages [5]. Considering the results from in vivo inhibition experiments, our study strongly suggests that repeated treatment with bortezomib causes caspase-dependent release of HMGB1 from macrophages, which in turn triggers neuronal sensitization by activating RAGE and accelerating CXCL12/CXCR4 signaling, resulting in CIPN (Figure 9).

The present study, together with a series of our studies on CIPN [2,4,5,6,29], ascertains that HMGB1 is a common crucial molecule responsible for the development of CIPN in rodents treated with different chemotherapeutics including bortezomib, paclitaxel, oxaliplatin, and vincristine. However, it is surprising that the origins of HMGB1 and molecular mechanisms for HMGB1 release vary depending on chemotherapeutics employed, e.g., the origins of HMGB1 are macrophages for bortezomib and paclitaxel [5] versus non-macrophage cells for oxaliplatin [6], and the mechanisms for HMGB1 release involve caspase for bortezomib versus ROS/p38MAPK/NF-κB signals for paclitaxel [5] (Figure 9). Our findings in inhibition experiments suggest that the extracellular HMGB1 derived from macrophages targets RAGE and CXCR4, but not TLR4 or TLR5, in the production of CIPN following bortezomib treatment in mice, being consistent with our previous study characterizing CIPN caused by paclitaxel in mice [5] (Figure 9). However, it is to be noted that spinal, but not peripheral, TLR4 might play a role in the CIPN caused by paclitaxel in C57BL/6 mice [5,30], and that the CIPN caused by oxaliplatin involves TLR4 in addition to RAGE and CXCR4 [6].

The present findings that repeated treatment with TMα completely prevented the CIPN following bortezomib treatment, as it did in rodents treated with paclitaxel, vincristine, or oxaliplatin [4,5,6], encourage its clinical application for the prevention of CIPN in various cancer patients undergoing different chemotherapeutic agents, in addition to colorectal cancer patients undergoing oxaliplatin-based chemotherapy [12]. The cancellation of the anti-CIPN effect of TMα by argatroban in bortezomib-treated mice (see Figure 2D) suggests its dependence on endogenous thrombin, as reported elsewhere [6,29,31]. Our results that repeated administration of argatroban significantly enhanced plasma HMGB1 levels and aggravated CIPN itself in mice treated with a subeffective dose of bortezomib (see Figure 2E,F), suggest the protective role of the endothelial TM/thrombin system as an HMGB1-inactivating system against CIPN, in agreement with our previous study using a mouse model of CIPN caused by oxaliplatin [6].

The links between macrophages, HMGB1, and CIPN do not necessarily explain the overall picture of molecular mechanisms underlying CIPN caused by bortezomib. In the present study, we found that HMGB1 levels in the sciatic nerve and plasma tended to increase only on day 14 after the onset of bortezomib treatment (see Figure 1D and Figure 2F), while macrophage accumulation in DRG was observed on day 3, but not day 14 (see Figure 5D–F). Thus, there are still some concerns to be addressed by further follow-up studies, such as the investigation of the effects of HMGB1 on CIPN in macrophage-depleted mice, monitoring of the time-course of changes in HMGB1 levels in the tissue and plasma.

Our findings that bortezomib as well as MG132, a well-known another proteasome inhibitor, directly stimulated the release of HMGB1 from mouse macrophage-like RAW264.7 cells (see Figure 6E) indicate that proteasome inhibition itself causes HMGB1 release in macrophages. There is evidence that HMGB1 can be released from macrophages during apoptosis [27,28], and that bortezomib induces apoptosis of cancer cells through stabilization and inhibition of proteasomal degradation of pro-apoptotic proteins such as p53 and IκB [32,33,34]. Therefore, it is understandable that bortezomib induced caspase activation and apoptosis in macrophages, leading to caspase-dependent HMGB1 release (see Figure 7D–I), and that the CIPN caused by bortezomib in mice was prevented by repeated administration of the caspase inhibitor (see Figure 8) in the present study. On the other hand, in our previous study [5], LPS-RS, AMD3100, and FPS-ZM1, antagonists of TLR4, CXCR4, and RAGE, respectively, that prevented the CIPN in paclitaxel-treated mice, did not inhibit the paclitaxel-induced HMGB1 release from macrophages. Nevertheless, considering the differences of molecular mechanisms for the release of HMGB1 from macrophages by bortezomib and paclitaxel, it is interesting to evaluate the involvement of autocrine or paracrine acceleration mechanisms in the bortezomib-induced HMGB1 release from macrophages in future.

Our results that the inactivation of HMGB1 by its neutralizing antibody or TMα reversed the established CIPN in mice treated with bortezomib (see Figure 1C and Figure 2B) suggest the role of HMGB1 in the maintenance of the CIPN, as reported in the CIPN caused by paclitaxel or oxaliplatin in rodents [4,6]. However, neither liposomal clodronate, a macrophage depletor, nor ethyl pyruvate, known to inhibit HMGB1 release from macrophages, had such effect on the established CIPN (see Figure 4F,G), suggesting that macrophages are not involved in the maintenance of CIPN. This is consistent with the detection of macrophage accumulation in the DRG on day 3, but not day 14, after the onset of bortezomib treatment (see Figure 5D–F). Nonetheless, minocycline, a macrophage/microglia inhibitor, reversed the established CIPN (see Figure 4E), as did the anti-HMGB1-neutralizing antibody or TMα (see Figure 1C and Figure 2B), suggesting a role of microglia in the maintenance of CIPN caused by bortezomib, although this has yet to be analyzed in-depth by future studies. Furthermore, the endothelium and nociceptors could be origins for the extracellular HMGB1, because the endothelial cells and nociceptive neurons secrete HMGB1 in response to inflammatory stimuli and/or following surgical injury to the sciatic nerves [35,36]. Our studies to clarify the involvement of HMGB1 derived from non-macrophage/non-microglial cells in CIPN are now in progress. Interestingly, blockers of RAGE, but not CXCR4, TLR4, or TLR5, also transiently reversed the established CIPN (see Figure 3B,F), which might be supported by our results that RAGE, but not TLR4 or CXCR4, in the DRG was upregulated on day 14 after the onset of bortezomib treatment (see Figure 3C), in agreement with the previous report concerning the bortezomib-induced upregulation of RAGE in the spinal cord [37]. However, the inhibition of the HMGB1/RAGE system in the persistent phase of CIPN following bortezomib might be quickly compensated by unknown pronociceptive systems, considering the short duration of the anti-CIPN effects of the anti-HMGB1-neutralizing antibody, TMα and RAGE antagonist (see Figure 1C, Figure 2B and Figure 3B).

## 5. Conclusions

In conclusion, the present study indicates that the activation or acceleration of RAGE and CXCR4 signals by macrophage-derived HMGB1 plays a crucial role in the development of CIPN in bortezomib-treated mice, as it did in paclitaxel-treated mice, which is negatively regulated by TM/TMα in a thrombin-dependent manner. However, the molecular mechanisms for the HMGB1 release from macrophages are different between bortezomib and paclitaxel, i.e., caspase-dependent apoptosis is involved in the HMGB1 release from macrophages and the development of CIPN caused by bortezomib, but not paclitaxel [5] (Figure 9). Thus, the present study sheds light on the previously unknown mechanisms for CIPN caused by bortezomib, including caspase-dependent HMGB1 release from macrophages, and justify pharmacological intervention targeting HMGB1 for the prevention of CIPN in cancer patients undergoing chemotherapeutics including bortezomib.

## Data Availability

The raw data are available from the corresponding author, upon request.

## Supplementary Materials

The following are available online at https://www.mdpi.com/article/10.3390/cells10102550/s1, Figure S1: Lack of effect of thrombomodulin α, FPS-ZM1, a RAGE antagonist, and Z-VAD-FMK, a pan-caspase inhibitor, on the nociceptive threshold in vehicle-treated mice.
