# Supplementary material for: Caspase-Dependent HMGB1 Release from Macrophages Participates in Peripheral Neuropathy Caused by Bortezomib, a Proteasome-Inhibiting Chemotherapeutic Agent, in Mice
Source: Cells. 2021 Sep 27;10(10):2550. doi: 10.3390/cells10102550 (PMC8533714; doi:10.3390/cells10102550)
Supplement: Supplementary file 1 [file cells-10-02550-s001.zip › cells-1345510-supplementary.pdf]

Supplementary Material

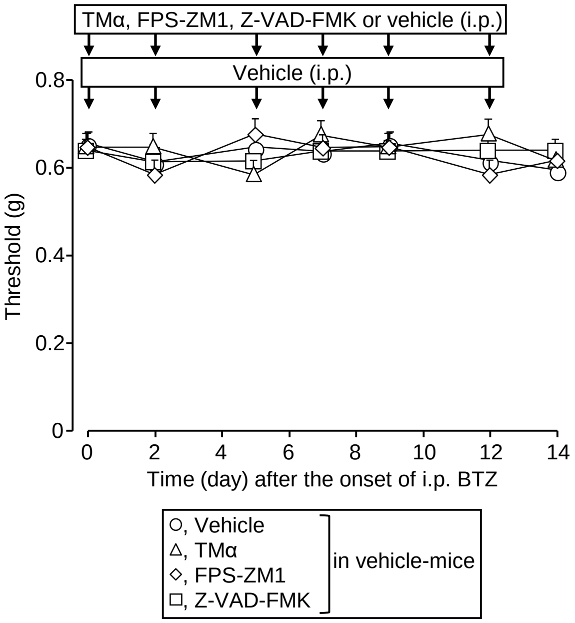

Figure S1. Lack of effect of thrombomodulin α, FPS-ZM1, a RAGE antagonist, and Z-VAD-FMK, a pan-caspase inhibitor, on the nociceptive threshold in vehicle-treated mice. Vehicle was administered i.p. on day 0, 2, 5, 7, 9 and 12. Thrombomodulin α at 10 mg/kg, FPS-ZM1 at 1 mg/kg or Z-VAD-FMK at 1 mg/kg was administered i.p. 30 min before each dose of vehicle. TMα, thrombomodulin α. Data show the mean with S.E.M. for 4-6 mice.
